# Angioedema Caused by Drugs That Prevent the Degradation of Vasoactive Peptides: A Pharmacovigilance Database Study

**DOI:** 10.3390/jcm10235507

**Published:** 2021-11-25

**Authors:** Yoshihiro Noguchi, Azusa Murayama, Hiroki Esaki, Mayuko Sugioka, Aisa Koyama, Tomoya Tachi, Hitomi Teramachi

**Affiliations:** 1 Laboratory of Clinical Pharmacy, Gifu Pharmaceutical University, 1-25-4, Daigakunishi, Gifu-shi 501-1196, Japan; 155074@gifu-pu.ac.jp (A.M.); 155040@gifu-pu.ac.jp (M.S.); 155034@gifu-pu.ac.jp (A.K.); tachi@gifu-pu.ac.jp (T.T.); 2 Department of Pharmacy, Ichinomiya Municipal Hospital, 2-2-22 Bunkyou, Ichinomiya-shi 491-8558, Japan; ichinomiyahp.esaki@gmail.com

**Keywords:** angiotensin-converting enzyme (ACE) inhibitors, dipeptidyl peptidase-4 (DPP-4) inhibitors, angioedema, Bayesian Confidence Propagation Neural Network (BCPNN), Japanese Adverse Drug Event Report (JADER) database

## Abstract

Angioedema results from the decreased degradation of vasoactive peptides such as substance P and bradykinin. In this study, we sought to clarify whether dipeptidyl peptidase-4 (DPP-4) and angiotensin-converting enzyme (ACE) inhibitors that suppress the degradation of substance P and bradykinin are involved in angioedema onset. We calculated information coefficients (ICs) by performing a disproportionality analysis to evaluate DPP-4/ACE inhibitor-induced angioedema using the Japanese Adverse Drug Event Report (JADER) database. No angioedema signals were detected for DPP-4 inhibitors; however, a signal was detected for ACE inhibitors (IC: 2.42, 95% confidence interval (CI): 2.19 to 2.65). Of the patients treated with DPP-4 inhibitors, four developed drug-induced angioedema in combination with ACE inhibitors, and all were taking vildagliptin. Signals were detected for enalapril (IC: 2.39, 95% CI: 2.06 to 2.71), imidapril (IC: 2.83, 95% CI: 2.38 to 3.27), lisinopril (IC: 2.28, 95% CI: 1.55 to 3.00), temocapril (IC: 1.35, 95% CI: 0.29 to 2.40), and trandolapril (IC: 1.57, 95% CI: 0.19 to 2.95). Both inhibitors inhibited the degradation of substance P and bradykinin and were thus expected to cause angioedema. However, no signal of angioedema was detected with the DPP-4 inhibitors, in contrast to some ACE inhibitors. This study found that ACE inhibitors and DPP-4 inhibitors, which inhibit the degradation of substance P and bradykinin, tended to have different effects on the onset of angioedema in clinical practice.

## 1. Introduction

Angioedema is characterized by localized deep dermal and subcutaneous mucosal edema caused by vasodilation and increased vascular permeability [1]. Drug-induced angioedema is best categorized as allergic or non-allergic, and the latter type develops as a consequence of the underlying mechanism of the drug. There are several causes of drug-induced angioedema, one being ACE inhibitor-induced angioedema. ACE inhibitor-induced angioedema may remit spontaneously, but many angioedemas relapse with the continued use of ACE inhibitors. Moreover, up to 16% of patients admitted for emergency treatment require tracheal intubation and 1% require tracheostomy [2].

**Figure 1:**
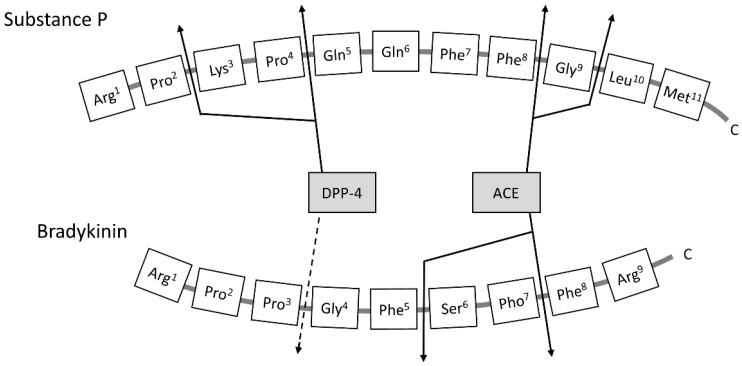
Sites of action of angiotensin-converting enzyme and dipeptidyl peptidase-4 in the metabolism of substance P and bradykinin.

ACE inhibitor-induced angioedema results from the decreased degradation of bradykinin and other vasoactive peptides such as substance P [3,4]. Substance P released from nerve endings upon stimulation of bradykinin increases vascular permeability by activating neurokinin 1 (NK1) receptors. Bradykinin and substance P are inactivated by ACE and dipeptidyl peptidase 4 (DPP-4) (Figure 1) [5].

Therefore, vasoactive peptide-induced angioedema may onset not only in ACE inhibitors but also in DPP-4 inhibitors. However, the contribution of impaired degradation of substance P by DPP-4 to the pathogenesis of vasoactive peptide-induced angioedema is unknown.

Many drug-induced adverse events (AEs) occur infrequently and may only be observed over the long term. Furthermore, the use of medicines in actual clinical settings post-marketing is complicated and, unlike in clinical trials, not restricted to a specific patient population. Therefore, determining the trends in the occurrence of AEs through the use of a large-scale database comprising long-term post-marketing data will contribute to the detection of early signals of and appropriate responses to AEs.

Safety signals based on the principle of disproportionality in the difference between the ratio of AEs reported are used as an index of detection. Safety signals can detect unknown AEs early, and numerous risk assessments have been reported [6,7,8,9,10,11]. 

There are several algorithms for signal detection [12,13,14]. One algorithm, a method based on Bayesian statistics (i.e., Bayesian Confidence Propagation Neural Network (BCPNN)), detects a stable signal even if the number of reports is small [15].

In this study, to clarify the effect of drugs that prevent the degradation of vasoactive peptides on the onset of angioedema, we assessed the safety signals using the Japanese Adverse Drug Event Report (JADER) database.

## 2. Materials and Methods

### 2.1. Data Source

This study used patient data contained in the JADER database, which was released in October 2019. It can be accessed directly here: http://www.info.pmda.go.jp/fukusayoudb/CsvDownload.jsp (in Japanese only), accessed on 1 October 2021.

**Figure 2:**
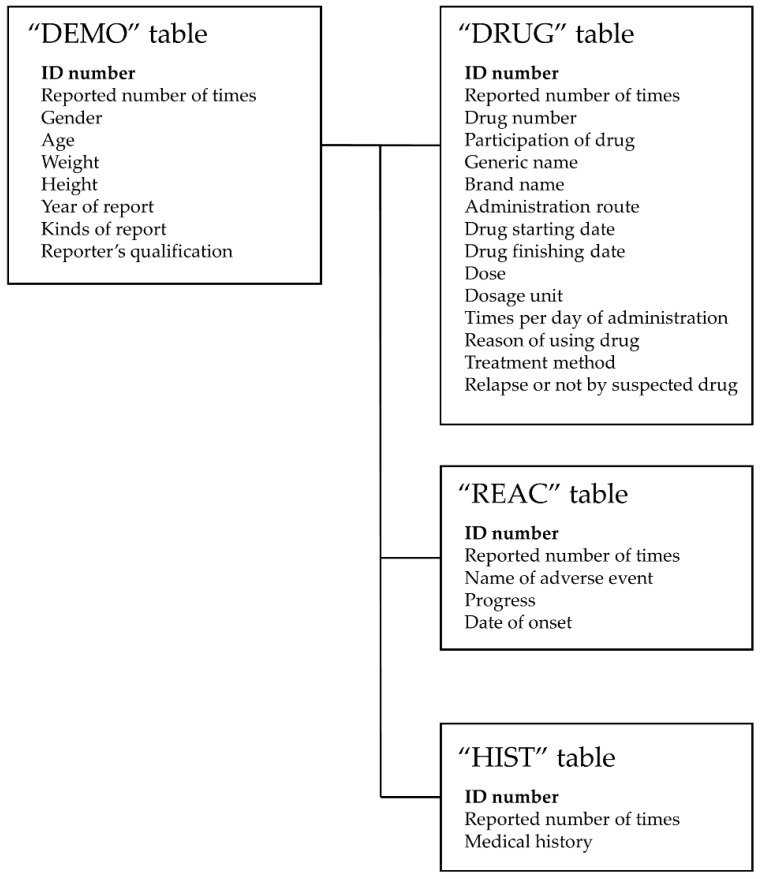
The four information tables included in JADER.

JADER comprises four tables of comma-separated values (csv) file format: DEMO.csv (patient information table), DRUG.csv (medicinal information table), HIST.csv (patient history table), and REAC.csv (AE information table) (Figure 2).

Generally, only cases of reported AEs, and not all cases of patients using the drug, are registered in the spontaneous reporting system. This means that this study using JADER has the same limitations as studies using other spontaneous reporting systems [14]. In addition, the unique features of JADER are as follows: (1) drugs approved in other countries are not necessarily approved in Japan; (2) as a post-marketing AE survey unique to Japan, it has been implemented to strengthen the information collection system for serious AEs for six months from the launch of new drugs; (3) the majority of those reporting to JADER are physicians (77.3%). The next most common reporters, pharmacists, comprise only 6.3%, while registration by lawyers is less than 0.01% [14].

### 2.2. Definitions of Suspected Drugs and Adverse Events

ACE inhibitors (12 drugs: alacepril, benazepril, captopril, cilazapril, delapril, enalapril, imidapril, lisinopril, perindopril, quinapril, temocapril, trandolapril) and DPP-4 inhibitors (9 drugs: alogliptin, anagliptin, linagliptin, omarigliptin, saxagliptin, sitagliptin, teneligliptin, trelagliptin, vildagliptin) were selected as the drugs to be investigated.

The AEs registered in the JADER database were the preferred terms used in the Medical Dictionary for Regulatory Activities/Japanese version (MedDRA/J) version 23.0. In this study, the targeted AEs were extracted from the preferred terms including angioedema (standardized MedDRA query (SMQ): 20000024 (Table S1)) in MedDRA standard search formula SMQ described in MedDRA/J. 

SMQ: 20000024 has narrow and broad scope terms, and both are used in this study. When creating the database for analysis, PT and SMQ were linked. If the PT associated with angioedema was registered with the same patient ID, it was processed so that it could be counted as one case. Therefore, the number of cases in which individual PTs registered for angioedema (shown in Table S1) cannot be counted.

In addition, the reported number of AEs due to the use of the target drugs was calculated based on the number of cases, and not the number of drug-AE combinations.

### 2.3. Signal Detection

Disproportionality analysis is based on the principle of disproportionality, which focuses on differences in the proportion of AE reports.

In this study, the signal obtained by the disproportionality analysis of suspected drug-induced angioedema was evaluated using the IC of the BCPNN, a method based on the Bayesian statistics model. The IC and 95% CI were calculated using Table 1 and Equations (1)–(4).

The IC_025_ value is the lower end of the 95% CI for IC. The detection criterion of this statistical model was IC_025_ > 0, similar to that used in the previous study [16].

(1) $$E\left(IC_{11}\right)=log_{2}\frac{\left(N_{11}+\gamma_{11}\right)\left(N_{++}+\alpha \right)\left(N_{++}+\beta \right)}{\left(N_{++}+\gamma \right)\left(N_{1+}+\alpha_{1}\right)\left(N_{+1}+\beta_{1}\right)}$$

(2) $$V\left(IC_{11}\right)={\left(\frac{1}{log2}\right)}^{2}\left[\frac{N_{++}-N_{11}+\gamma -\gamma_{11}}{\left(N_{11}+\gamma_{11}\right)\left(1+N_{++}+\gamma \right)}+\frac{N_{++}-N_{1+}+\alpha -\alpha_{1}}{\left(N_{1+}+\alpha_{1}\right)\left(1+N_{++}+\alpha \right)}+\frac{N_{++}-N_{+1}+\beta -\beta_{1}}{\left(N_{+1}+\beta_{1}\right)\left(1+N_{++}+\beta \right)}\right]$$

(3) $$\gamma =\gamma_{11}\frac{\left(N_{++}+\alpha \right)\left(N_{++}+\beta \right)}{\left(N_{1+}+\alpha_{1}\right)\left(N_{+1}+\beta_{1}\right)},\;\gamma_{11}=1,\;\alpha_{1}=\beta_{1}=1,\;\alpha =\beta =2$$

(4) $$IC\;\left(95\%\;\mathrm{confidence}\;\mathrm{interval}\right)=E\left(IC_{11}\pm 2\sqrt{V\left(IC_{11}\right)}\right)$$

Upper limits of the 95% CI for IC (= IC_975_) of <0 signified inverse associations [17,18,19]. That is, these associations were explored as the inverse signals. However, determining that the inverse signals are a therapeutic effect rather than a side effect requires a more detailed study. In previous studies, inverse signals were verified through other database studies [17,18] and animal experiments [19].

## 3. Results

After excluding reports that used subjective terms such as “youth” and “elderly”, as well as those without information about sex and age, 534,287 patient reports (all cases) were obtained. Among all cases, ACE inhibitors and DPP-4 inhibitors were used in 1578 and 6898 cases, respectively. Table 2 shows the characteristics of patients using ACE inhibitors/DPP-4 inhibitors and their distribution by sex and age.

**Figure 3:**
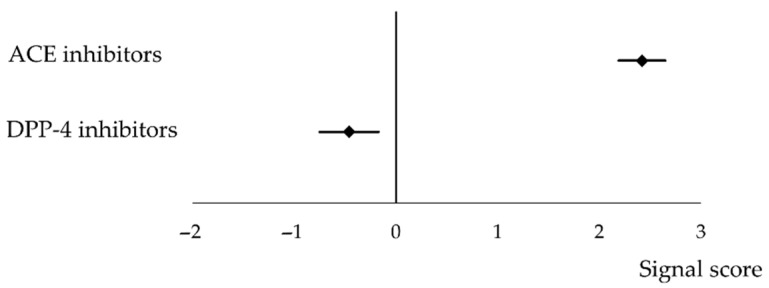
The signal scores of angiotensin-converting enzyme inhibitor and dipeptidyl peptidase-4 inhibitor-induced angioedema.

Figure 3 shows the signal score (information components, ICs) of ACE inhibitor/DPP-4 inhibitor-induced angioedema.

### 3.1. ACE Inhibitor-Induced Angioedema

A signal was detected (IC: 2.42, 95% CI: 2.19–2.65) in 176 cases of ACE inhibitor-induced angioedema (Figure 3, Table 3).

In addition, when ACE inhibitors were analyzed, signals were detected for enalapril (*N*_11_: 86, IC: 2.39, 95% CI: 2.06–2.71), imidapril (*N*_11_: 48, IC: 2.83, 95% CI: 2.38–3.27), lisinopril (*N*_11_: 17, IC: 2.28, 95% CI: 1.55–3.00), temocapril (*N*_11_: 7, IC: 1.35, 95% CI: 0.29–2.40), and trandolapril (*N*_11_: 4, IC: 1.57, 95% CI: 0.19–2.95) (Table 3).

### 3.2. DPP-4 Inhibitor-Induced Angioedema

One hundred and one cases of angioedema associated with DPP-4 inhibitors were reported, and no signal was detected. However, the inverse signals detected were: total (IC: −0.46, 95% CI: −0.75 to −0.17); sitagliptin (*N*_11_: 26, IC: −0.71, 95% CI: −1.27 to −0.15); and teneligliptin (*N*_11_: 4, IC: −1.37, 95% CI: −2.66 to −0.07). (Figure 3, Table 4).

Of the reported cases of DPP-4 inhibitor-induced angioedema, four were in combination with an ACE inhibitor. In all cases, vildagliptin was used (Table 5).

## 4. Discussion

Vasoactive peptide-induced angioedema may onset not only in ACE inhibitors but also in DPP-4 inhibitors. However, the contribution of impaired degradation of substance P by DPP-4 to the pathogenesis of vasoactive peptide-induced angioedema is unknown. This study was conducted using the JADER database to clarify the effect of drugs that inhibit the degradation of vasoactive peptides on the onset of angioedema.

Signals of angioedema for the following ACE inhibitors (total) (IC_025_: 2.19) were detected: enalapril (IC_025_: 2.06), imidapril (IC_025_: 2.38), lisinopril IC_025_: 1.55), temocapril (IC_025_: 0.29), and trandolapril (IC_025_: 0.19) (Table 3).

These results are consistent with the onset mechanism of angioedema and further indicate that the spontaneous reporting system can be used to detect the signal of ACE inhibitor-induced angioedema (vasoactive peptide-induced angioedema). However, in this study, not all ACE inhibitors detected a signal. This may be due to differences in the use of each ACE inhibitor in Japan, which may have affected the number of AEs reported.

Evidence from previous studies implicates DPP-4 deficiency in the pathogenesis of vasoactive peptide-induced angioedema. Byrd et al. found that rats genetically deficient in DPP-4 were more likely to have increased tracheal edema after the administration of ACE inhibitors, and that effect was blocked by substance P [20]. It has also been reported that some individuals who experience vasoactive peptide-induced angioedema have lower serum DPP-4 enzyme activity than control subjects [21]. This suggests that vasoactive peptide-induced angioedema may result from impaired substance P degradation by DPP-4 inhibitors.

On the other hand, spontaneous reports (including post-marketing reports) have been reported but, so far, epidemiological studies have not demonstrated an increased risk of DPP-4 inhibitors in a lone induced angioedema [5]. 

In this study, no signal for DPP-4 inhibitors was detected. Instead, the following inverse signals were detected: total (IC_975_: −0.17), sitagliptin (IC_975_: −0.15), teneligliptin (IC_975_: −0.07) (Table 4). The inverse signals detected for the DPP-4 inhibitors are contrary to the results expected from the pharmacological mechanism.

The study design using the spontaneous reporting system can generate hypotheses such as signals (inverse signals), but it does not thoroughly test those signals (inverse signals). Whether or not inverse signals have a therapeutic effect requires analysis from multiple perspectives. Several previous studies related to inverse signals include analysis using claims databases as real-world data [17,18] and animal experiments [19]. However, this study design uses only JADER, as only the signal (inverse signal) is explored. More detailed clinical studies are needed to convert the signals (inverse signals) into higher evidence [14].

On the other hand, apart from this study, it has been shown that the pharmacologically assumed impairment of substance P degradation by DPP-4 inhibitors may have little clinical impact. An example was provided in a previous research paper [10]. Although DPP-4 inhibitors were expected to prevent dysphagia and aspiration pneumonia because they prevent the degradation of substance P involved in the swallowing reflex, the previous study revealed that DPP-4 inhibitors were strongly associated with onset rather than preventing aspiration pneumonia.

Thus, the amount of DPP-4 inhibitor used in clinical practice may be insufficient to suppress the degradation of substance P. However, the database used in this study had only clinical usage data, and it was impossible to thoroughly verify the relationship between DPP-4 inhibitors and substance P levels. In future, it will be necessary to verify our hypothesis using real-world data rather than a spontaneous reporting system.

This study suggests that the use of ACE inhibitors and DPP-4 inhibitors, which inhibit the degradation of substance P and bradykinin, in the clinic tend to have different effects on the onset of vasoactive peptide-induced angioedema. It has also been reported that under conditions of ACE inhibition, DPP-4 plays a significant role in the degradation and inactivation of substance P. Brown et al. reported that concurrent DPP-4 inhibitor and ACE inhibitor use increases the risk of vasoactive peptide-induced angioedema [5]. Although there were only four cases in this study, they were similar to a previous study [5]. Furthermore, signals of angioedema with the combination of ACE and DPP-4 inhibitors have been reported in research using the WHO pharmacovigilance database [22]. Thus, DPP-4 inhibitor-induced angioedema should be carefully considered and monitored, especially during concurrent treatment with ACE inhibitors.

Although cases registered in the JADER database include data on post-marketing surveillance, they are mainly spontaneous reports. Therefore, only a proportion of the AEs recognized in clinical practice have been included in the database. In addition, there are also several reporting biases (Weber effect: the number of reported AEs after post-marketing decreases over time following an immediate transient increase [23]; notoriety effects: the number of reported AEs on a topic increase overall [24]; masking effect: numerous reports associating the same AEs with other drugs lead to the signal being underestimated [25]). Therefore, the signals (inverse signals) must be interpreted carefully [14].

Many studies utilize reporting odds ratio (ROR) to detect signals in the signal detection study because it is easy to calculate. However, such methods do not provide stable signals when the number of reports is small [15]. In this study, signals were calculated for each ACE inhibitor and DPP-4 inhibitor. Of them, few reported as DPP-4 inhibitors. Therefore, we evaluated using BCPNN, which provides stable signals even if the number of reports is small.

Although our findings need further validation and should be interpreted cautiously given the study’s limitations, it is necessary to understand that even though the mechanism of the onset of angioedema by each drug was similar, the scores of the signals were very different.

## 5. Conclusions

In this study, we found that ACE inhibitors and DPP-4 inhibitors that inhibit the degradation of substance P and bradykinin tend to have different effects on the onset of angioedema in clinical practice. Longitudinal observational research is needed to fully understand the association between ACE inhibitor/DPP-4 inhibitor use and vasoactive peptide-induced angioedema. Physicians should be aware of possible associations from this study.

## Figures and Tables

**Table 1:** The 2 × 2 contingency table for signal detection.

	Target AEs	Other AEs	Total
Target drug	*N*_11_	*N*_10_	*N*_1+_
Other drugs	*N*_01_	*N*_00_	*N*_0+_
Total	*N*_+1_	*N*_+0_	*N*_++_

*N*: the number of reports; AE: adverse event.

**Table 2:** Characteristics of patients using ACE inhibitors/DPP-4 inhibitors and their distribution by sex and age.

	ACE Inhibitors			DPP-4 Inhibitors		
Age	*N*_11_	*N*_1+_	RR (%)	*N*_11_	*N*_1+_	RR (%)
Total	176	1578	11.2	101	6898	1.5
Female	60	661	9.1	44	2739	1.6
Male	116	917	12.6	57	4159	1.4
<40	5	173	2.9	1	83	1.2
40–49	3	60	5.0	7	269	2.6
50–59	19	163	11.7	21	645	3.3
60–69	48	333	14.4	22	1659	1.3
70–79	57	501	11.4	29	2342	1.2
80–89	43	299	14.4	18	1634	1.1
≥90	1	49	2.0	3	266	1.1

Legend: ACE inhibitors: angiotensin-converting enzyme inhibitors; DPP-4 inhibitors: dipeptidyl peptidase-4 inhibitors; *N*_11_: the number of target-drug-induced angioedema; *N*_1+_: the number of all target-drug-induced adverse events; RR: reporting rate (= *N*_11_/*N*_1+_).

**Table 3:** The signal scores of each angiotensin-converting enzyme (ACE) inhibitor.

Drug	*N*_11_	*N*_1+_	IC (95% CI)
ACE inhibitors	176	1578	2.42 * (2.19–2.65)
Alacepril	3	21	1.47 (−0.10–3.03)
Benazepril	1	13	0.64 (−1.54–2.82)
Captopril	2	63	0.39 (−1.32–2.09)
Cilazapril	1	12	0.66 (−1.53–2.85)
Delapril	0	10	−0.29 (−3.30–2.72)
Enalapril	86	771	2.39 * (2.06–2.71)
Imidapril	48	291	2.83 * (2.38–3.27)
Lisinopril	17	133	2.28 * (1.55–3.00)
Perindopril	6	114	1.07 (−0.05–2.20)
Quinapril	1	30	0.30 (−1.81–2.40)
Temocapril	7	105	1.35 * (0.29–2.40)
Trandolapril	4	33	1.57 * (0.19–2.95)

Legend: ACE: angiotensin-converting enzyme; *N*_11_: the number of ACE inhibitor-induced angioedema; *N*_1+_: the number of all ACE inhibitor-induced adverse events; IC: the information components; CI: confidence interval; *: signal.

**Table 4:** The signal scores of each dipeptidyl peptidase-4 (DPP-4) inhibitor.

Drug	*N*_11_	*N*_1+_	IC (95% CI)
DPP-4 inhibitors	101	6898	−0.46 ^†^ (−0.75–−0.17)
Alogliptin	15	633	0.21 (−0.52–0.94)
Anagliptin	2	212	−0.82 (−2.50–0.85)
Linagliptin	8	736	−0.82 (−1.79–0.14)
Omarigliptin	0	131	―
Saxagliptin	1	224	−1.47 (−3.52–0.58)
Sitagliptin	26	2131	−0.71 ^†^ (−1.27–−0.15)
Teneligliptin	4	587	−1.37 ^†^ (−2.66–−0.07)
Trelagliptin	4	166	0.19 (−1.12–1.50)
Vildagliptin	45	2183	0.02 (−0.41–0.46)

Legend: DPP-4: dipeptidyl peptidase-4; *N*_11_: the number of DPP-4 inhibitor-induced angioedema; *N*_1+_: the number of all DPP-4 inhibitor-induced adverse events; IC: the information components; CI: confidence interval; ^†^: inverse signal.

**Table 5:** Data of patients on the concomitant use of DPP-4 inhibitors and ACE inhibitors.

Patient ID	Sex	Age	DPP-4 Inhibitors	ACE Inhibitors	Report Year
AB-11007250	male	60s	vildagliptin	perindopril	2011
AB-11029595	male	70s	vildagliptin	enalapril	2011
AB-11040470	male	60s	vildagliptin	enalapril	2011
AB-12027781	male	70s	vildagliptin	enalapril	2012

Legend: 60s: 60–69 years old; 70s: 70–79 years old.

## Data Availability

This study used patient data contained in the JADER database, which was released in October 2019 (in Japanese only). However, the Japanese authority which owns this data (the PMDA) does not permit sharing the data directly. It can be accessed directly here: http://www.info.pmda.go.jp/fukusayoudb/CsvDownload.jsp (accessed on 1 October 2021).

## Supplementary Materials

The following are available online at https://www.mdpi.com/article/10.3390/jcm10235507/s1. Table S1: The preferred term (PT) and code included in standardized MedDRA query (SMQ): 20000024.
