# Supplementary material for: Angioedema Caused by Drugs That Prevent the Degradation of Vasoactive Peptides: A Pharmacovigilance Database Study
Source: J Clin Med. 2021 Nov 25;10(23):5507. doi: 10.3390/jcm10235507 (PMC8658484; doi:10.3390/jcm10235507)
Supplement: Supplementary file 1 [file jcm-10-05507-s001.zip › jcm-1453190-Supplementary.pdf]

Table S1. The preferred term (PT) and code included in standardized MedDRA query (SMQ): 20000024.

**angioedema (SMQ: 20000024)**			
**Narrow scope terms**		**Broad scope terms**	
code	PT	code	PT
10081035	Acquired C1 inhibitor deficiency	10003800	Auricular swelling
10060934	Allergic oedema	10006294	Breast oedema
10002424	Angioedema	10006312	Breast swelling
10052250	Circumoral oedema	10008589	Choking
10081703	Circumoral swelling	10008590	Choking sensation
10010726	Conjunctival oedema	10013700	Drug hypersensitivity
10011033	Corneal oedema	10014025	Ear swelling
10015029	Epiglottic oedema	10067450	Endotracheal intubation
10052139	Eye oedema	10058061	Gastrointestinal oedema
10015967	Eye swelling	10018092	Generalised oedema
10015993	Eyelid oedema	10067639	Genital swelling
10016029	Face oedema	10020751	Hypersensitivity
10049305	Gingival oedema	10052390	Laryngeal dyspnoea
10018291	Gingival swelling	10059639	Laryngeal obstruction
10066837	Gleich's syndrome	10048961	Localised oedema
10019860	Hereditary angioedema	10028748	Nasal obstruction
10080955	Hereditary angioedema with C1 esterase inhibitor deficiency	10028750	Nasal oedema
10073257	Idiopathic angioedema	10059012	Nipple oedema
10021247	Idiopathic urticaria	10058680	Nipple swelling
10076229	Intestinal angioedema	10061877	Obstructive airways disorder
10023845	Laryngeal oedema	10030095	Oedema
10023893	Laryngotracheal oedema	10080039	Oedema blister
10070492	Limbal swelling	10030104	Oedema genital
10024558	Lip oedema	10030111	Oedema mucosal
10024570	Lip swelling	10061317	Oedema neonatal
10075203	Mouth swelling	10030124	Oedema peripheral
10067317	Oculorespiratory syndrome	10031051	Orbital oedema
10030110	Oedema mouth	10066774	Penile oedema
10078783	Oropharyngeal oedema	10034319	Penile swelling
10031118	Oropharyngeal swelling	10078818	Perinephric oedema
10056998	Palatal oedema	10049779	Peripheral oedema neonatal
10074403	Palatal swelling	10048959	Peripheral swelling
10034545	Periorbital oedema	10062109	Reversible airways obstruction
10056647	Periorbital swelling	10039755	Scrotal oedema
10034829	Pharyngeal oedema	10039759	Scrotal swelling
10082270	Pharyngeal swelling	10058679	Skin oedema
10057431	Scleral oedema	10053262	Skin swelling
10042682	Swelling face	10076991	Soft tissue swelling
10042690	Swelling of eyelid	10042241	Stridor
10042727	Swollen tongue	10042444	Suffocation feeling
10043967	Tongue oedema	10042674	Swelling
10044296	Tracheal oedema	10079645	Therapeutic product cross-reactivity
10046735	Urticaria	10043528	Throat tightness
10046740	Urticaria cholinergic	10044291	Tracheal obstruction
10052568	Urticaria chronic	10044320	Tracheostomy
10046750	Urticaria papular	10045240	Type I hypersensitivity
		10067775	Upper airway obstruction
		10063818	Vaginal oedema
		10065768	Visceral oedema
		10047763	Vulval oedema
		10071211	Vulvovaginal swelling
		10047924	Wheezing

PT: preferred term.
